# Enhancing multisensory rehabilitation of visual field defects with transcranial direct current stimulation: A randomized clinical trial

**DOI:** 10.1111/ene.16559

**Published:** 2024-11-28

**Authors:** Lorenzo Diana, Carlotta Casati, Lisa Melzi, Stefania Bianchi Marzoli, Nadia Bolognini

**Affiliations:** ^1^ Department of Neurorehabilitation Sciences, Laboratory of Neuropsychology IRCCS Istituto Auxologico Italiano Milan Italy; ^2^ Neuro‐Ophthalmology Center and Ocular Electrophysiology Laboratory IRCCS Istituto Auxologico Italiano Milan Italy; ^3^ Department of Psychology University of Milano‐Bicocca and NeuroMI Milan Italy

**Keywords:** hemianopia, rehabilitation, stroke, transcranial direct current stimulation

## Abstract

**Background and Purpose:**

Visual rehabilitation is necessary for improving the quality of life of patients with acquired homonymous visual field defects (HVFDs). By modulating brain excitability and plasticity, transcranial direct current stimulation (tDCS) may accelerate and increase the effects of compensatory trainings, which are usually long and intensive. In the present proof‐of‐principle, double‐blind, randomized, sham‐controlled study, we assess whether anodal tDCS applied over ipsilesional occipital or parietal cortices can increase the effects of a compensatory audiovisual training for HVFDs.

**Methods:**

Eighteen participants with chronic HVFDs were randomized to receive anodal or sham tDCS over the ipsilesional parietal or occipital cortex during a 2‐week (10 days, 2 h/day) audiovisual treatment aimed at improving oculomotor visual field exploration. Improvements were assessed by administering visual detection with eye movements and visual search tests, and a questionnaire for activities of daily living (ADLs) before the treatment, at its end, and at 1‐month and 4‐month follow‐ups; lesion analyses were performed to look for predictors of treatment effects.

**Results:**

Anodal ipsilesional tDCS, regardless of the target area (occipital vs. parietal), speeds up and increases daily improvements during the training. Whereas long‐lasting (up to 4 months) post‐treatment improvements in visual search and ADLs were observed in all groups, a greater and stable increase of visual detections in the blind hemifield was brought about only by the adjuvant use of occipital tDCS.

**Conclusions:**

Compensatory audiovisual rehabilitation of HFVDs is effective and benefits from the adjuvant application of occipital and parietal tDCS, which speeds up and increases training‐induced improvement.

**Registry number: NCT06116760.:**

## INTRODUCTION

Homonymous visual field defects (HVFDs) after acquired brain injuries can significantly hinder daily activities, negatively impacting the quality of life of those affected [1, 2, 3]. Rehabilitation for HVFDs is based on restitutive and compensatory strategies [4, 5]. The former aim at enlarging the visual field [6, 7] through the intensive stimulation of the so‐called transition zone; compensatory therapies, instead, train oculomotor visual field scanning to overcome the visual field loss [6, 7, 8, 9], thus reducing its impact on everyday life activities [10, 11].

Noninvasive brain stimulation, such as transcranial direct current stimulation (tDCS), can be used to enhance the effectiveness of poststroke visual rehabilitation [12, 13, 14]. So far, anodal tDCS of the injured occipital cortex was typically combined with restituive trainings [15, 16, 17, 18, 19]. Despite some encouraging findings, a Level C recommendation has been assignied to this approach [12]. The adjuvant role of tDCS in compensatory treatments has not yet been explored.

Our study aims to assess whether tDCS can increase the effects of compensatory training for HVFDs. As compensatory treatment, we chose the audiovisual training (AVT), whose clinical efficacy has been widely documented [20, 21, 22, 23, 24, 25]. AVT is thought to activate multisensory integration mechanisms subserved by a spared retinocollicular–extrastriate pathway, improving visual exploration [26, 27].

In the present proof‐of‐principle, double‐blind, randomized study, brain‐damaged adults with acquired HVFDs underwent a 2‐week AVT combined with real or sham tDCS delivered over the ipsilesional occipital or parietal cortex [28]. Specifically, we aimed to assess whether and what tDCS protocol can accelerate and/or increase the effectiveness of the compensatory training, promoting longer lasting improvements of oculomotor visual field scanning (primary outcome) and vision‐related functional disability, also exploring predictors of treatment efficacy and its neuroanatomical correlates.

## METHODS

### Participants

**FIGURE 1:**
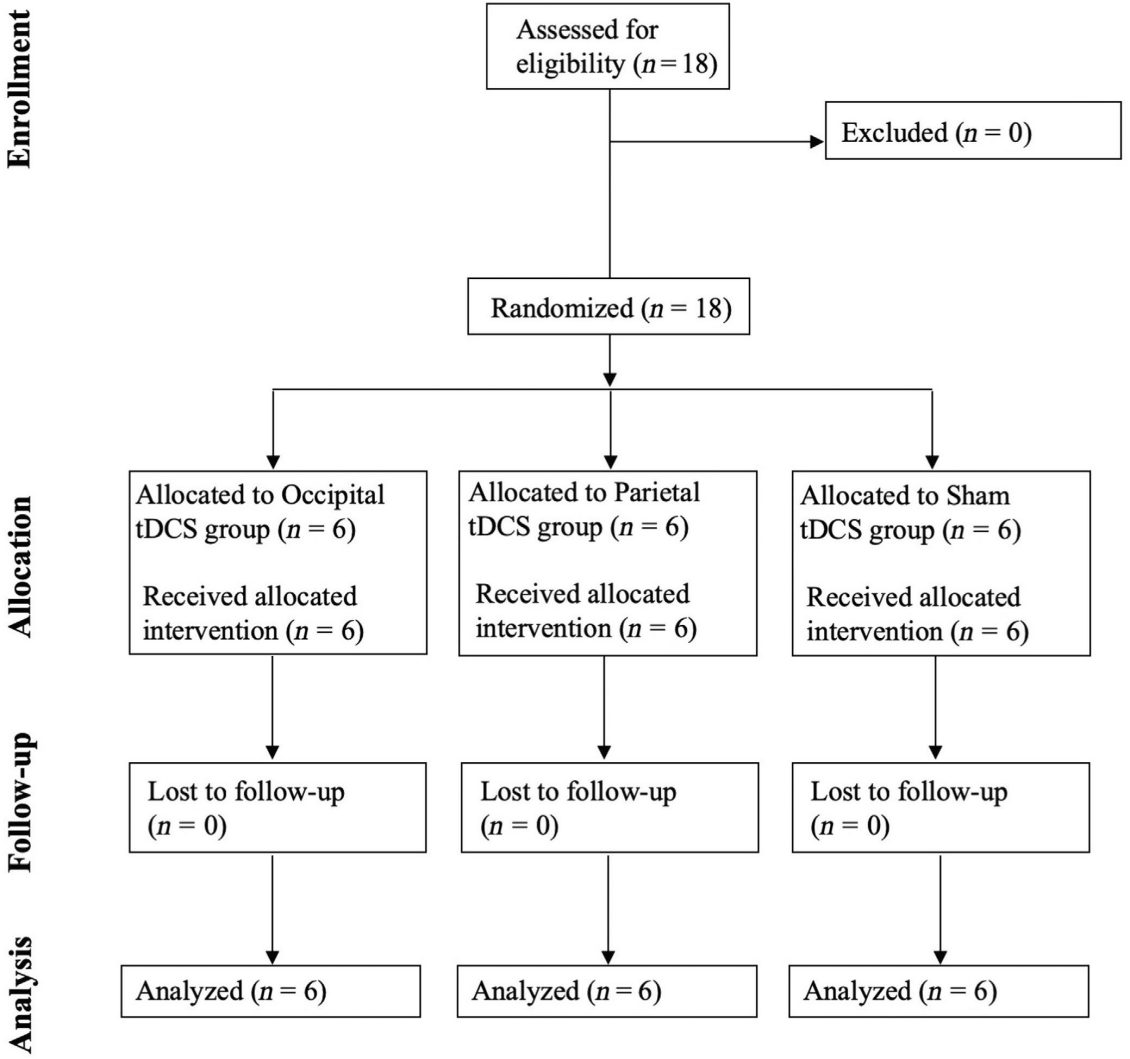
CONSORT (Consolidated Standards of Reporting Trials) flow diagram. tDCS, transcranial direct current stimulation.

According to a power analysis (see Appendix S1), 18 brain‐damaged individuals (mean age = 54 ± 13 SD years, range = 28–73 years) with chronic HVFDs (median disease duration = 248 days, interquartile range = 195 days, range = 90–2243 days) were recruited (see Figure 1) at the Neuro‐Ophthalmology Center and Ocular Electrophysiology Laboratory and at the Laboratory of Neuropsychology of the IRCSS Istituto Auxologico Italiano hospital (Milan, Italy).

The presence of HVFDs was documented by means of monocular computerized perimetry of both eyes (Humphrey Visual Field Analyzer, 30‐2, SITA standard; Participant P08 underwent standard Goldman perimetry). All patients had no hearing deficits and did not present counterindications to tDCS [29]. Clinical and demographic details are reported in Table 1.

**TABLE 1:** Demographic and clinical data.

tDCS	ID	Age, years	Sex	Disease duration, days	Etiology	Lesion	VF size^a^	HVFD
Ipsilesional occipital cortex	P01	52	M	181	I	Left, T‐O	−1.41	Right HH
	P02	57	M	239	I	Left, mesial O	−10.44	Right HH
	P03	67	M	339	I	Left, T‐O	−7.36	Right Sup Quad
	P04	64	F	181	H	Right, P‐O	−20.81	Left HH
	P05	31	F	297	TBI	Right, axonal damage	−17.16	Left HH
	P06	39	M	110	I	Right, mesial T, O, Thal	−13.05	Left HH
Ipsilesional parietal cortex	P07	67	M	130	I	Left, T‐O, Thal	−12	Right HH
	P08	60	M	402	H	Left, P‐O	Other^b^	Right Sup Quad
	P09	69	M	562	H	Left, T–P‐O	−11	Right HH
	P10	54	M	196	TBI	Left, Fr basal; Right, Fr‐P; axonal damage	−10.72	Right HH
	P11	28	M	2243	AVM	Left, O	−12.6	Right HH
	P12	46	M	90	I	Right, mesial, Thal	−6.4	Left Sup Quad
Sham	P13	66	M	400	I	Left, T‐O	−7.87	Right Sup Scot
	P14	45	M	258	I	Left, T‐O‐P	−14.11	Right HH
	P15	73	F	387	H	Left, P‐O	−10.2	Right HH
	P16	53	M	258	H	Right, posterior	−15.36	Left HH
	P17	43	M	154	I	Right, O	−14.9	Left HH
	P18	57	M	180	I	Right, Thal‐Ins, medial T	−7.7	Left Sup Quad

Abbreviations: AVM, arteriovenous malformation; F, female; Fr, frontal; H, hemorrhagic stroke; HH, homonymous hemianopia; HVFD, homonymous visual field defect; I, ischemic stroke; Ins, insular; M, male; O, occipital; P, parietal; Sup Quad, superior quadrantanopia; Sup Scot, superior scotoma; T, temporal; TBI, traumatic brain injury; tDCS, transcranial direct current stimulation; Thal, thalamic; VF, visual field.

^a^ Average mean deviation (in decibel, db) of Humphrey's monocular visual field analysis of the left and the right eye, 30‐2, Swedish Interactive Thresholding Algorithm standard.

^b^ P08 had been assessed with Goldman perimetry.

One week before the beginning of the training, all participants underwent a neuropsychological evaluation of visual field scanning abilities. No signs of hemispatial neglect were detected through line bisection, copy of Gainotti's figure [30], letter, and star cancellation tasks [31].

Participants were randomly assigned (random sequence generation, enrollment, and allocation carried out by C.C.) to one of three possible groups (i.e., tDCS protocols): ipsilesional occipital tDCS, ipsilesional parietal tDCS, or sham tDCS (see below).

The study, registered on clinicaltrials.gov (NCT06116760), was approved by the local ethics committee (protocol ID: 25C901_2009) and was conducted in accordance with the principles of the Declaration of Helsinki. All patients provided their written informed consent.

### Lesion mapping

Lesion maps could be reconstructed for 15 of 18 participants; at the time of the experiment, P05's and P10's magnetic resonance imaging showed only signs of diffused axonal injury that could not be mapped, whereas no recent imaging was available for P16.

**FIGURE 2:**
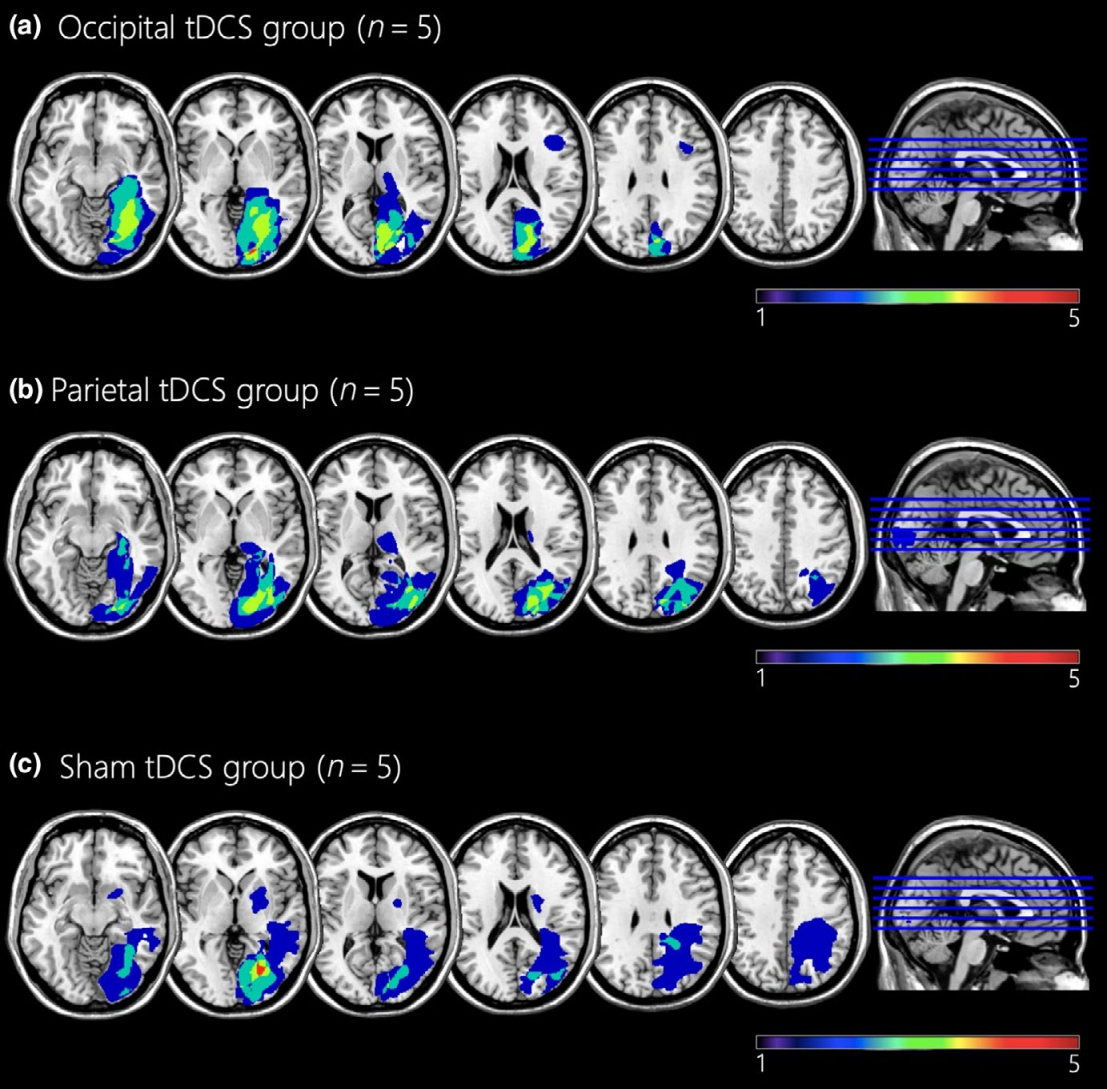
Lesion localization of brain‐damaged patients. Overlay lesion plots (frequencies of overlapping lesions, from dark blue, i.e., minimum overlap, to red, i.e., maximum overlap) for (a) the occipital transcranial direct current stimulation (tDCS) group, (b) the parietal tDCS group, and (c) the sham tDCS group. The mean lesion volume was 36.5 ± 31.5 cm^3^ (range = 3.95–127 cm^3^). The most affected areas, irrespective of the lesion side, were the calcarine sulcus (*n* = 15), the lingual gyrus (*n* = 15), the superior (*n* = 12), the middle (*n* = 12), and the inferior (*n* = 11) occipital lobes, as well as the cuneus (*n* = 9), and the fusiform gyrus (*n* = 12). Moreover, a number of intra‐ and interhemispheric white matter bundles were affected: corpus callosum (*n* = 15), inferior fronto‐occipital fasciculus (*n* = 15), inferior longitudinal fasciculus (*n* = 15), optic radiation (*n* = 14), posterior cingulum bundle (*n* = 12), and the first and second branches of the superior longitudinal fasciculus (*n* = 9). Left‐hemispheric lesions were mirrored onto the right hemisphere (neurological convention).

The lesion mapping procedure consisted in the analysis of lesion volume and its location including injured white matter tracts as in Diana et al. [28], which is detailed in the Appendix S1. Lesion map overlays are depicted in Figure 2.

### Audiovisual training

The training board consisted of a central 1 × 2 m part, with two 2 × 0.5 m side wings tilted 45° inward; 48 red light‐emitting diodes (LEDs; diameter = 1 cm, luminance = 90 cd/m^2^) arranged in six horizontal rows (eight lights per row) were distributed on the board; 48 piezoelectric speakers (0.4 W, 8 Ω; auditory stimulus = 80 dB) were arranged at the LEDs (EMS srl, www.emsmedical.net). During the training, spatiotemporally coincident audiovisual stimuli (100 ms) were randomly presented, one at a time, at one of 44 locations (22 locations for each hemifield). Six catch trials (no visual or auditory stimuli) were also presented. Participants were instructed to look at the central fixation point (2°) and move their eyes toward the visual stimulus, ignoring the concurrent auditory ones (the auditory stimulus aided visual detection thanks to multisensory integration, but was not the target [32]), and then report the detection of the visual stimulus by pressing the button of a wireless mouse [23]. The experimenter started the next trial only after the participant's eyes returned to the central fixation point (for details, see Diana et al. [28] and Bolognini et al. [33]).

The AVT consisted of 10 daily sessions (Monday to Friday, 2 h/day) during which blocks of 204 audiovisual trials were administered (three trials per spatial location in the sighted hemifield and six trials in the blind hemifield; average block duration = 15 min). In each session, the number of blocks varied according to the participant's speed or fatigue.

### tDCS protocol

During each daily training session, tDCS was delivered by a battery‐driven current stimulator (BrainStim; http://www.emsmedical.net) through a couple of rubber‐conductive electrodes (25 cm^2^) inserted into saline‐soaked sponges, kept in place with rubber bands. Anodal tDCS was applied at an intensity of 2 mA during the first 30 min of the AVT; for sham tDCS, the stimulator was turned off after 30 s [34]. The protocol was double‐blind: participants and experimenters were both kept blind to the tDCS condition (real or sham).

Participants were randomized to receive real or sham tDCS over the occipital (i.e., O1/O2 of the 10–20 electroencephalographic system) or posterior parietal (i.e., P03/P04) cortex of the ipsilesional hemisphere, with the cathode placed contralaterally, in a supraorbital position. All participants well tolerated tDCS and reported no adverse side effects.

### Assessment of the training effects

Treatment effects were assessed (i) by measuring day‐by‐day improvements during the AVT and (ii) by administering a battery of tests, assessing visual search and visual detections with eye movements [35], before the beginning of the treatment (Pre, i.e., baseline), immediately at the end of the training (Post), and at 1 and 4 months after its completion (follow‐ups, FU1 and FU4); activities of daily living (ADLs) were measured only before the AVT and at the FUs [36], as we expected that treatment benefits in daily living would consolidate over a longer period of time after the end of the treatment.

#### Visual detection task with eye movements

The apparatus used during training was also used to present 88 visual‐only targets (100 ms) in random order at one of 44 spatial positions (22 locations for each hemifield; two trials per spatial position), along with 12 catch trials (no visual stimulus). Participants were asked to press the button of a wireless mouse whenever they detected the visual stimulus. During the task, eye movements were allowed to detect the visual stimulus. At the end of each trial, the experimenter ensured—by visually checking—that the participant returned to the central fixation before presenting a new trial.

#### Visual search tests

Two tasks were used to assess visual scanning: the EF test and the Triangles test [23]. In both tests, stimulus matrices were projected onto a wall (SONY‐VPL‐ES4 projector), at a distance of 1.2 m (visual angle = 35° × 28°). Stimuli presentation and response recording were controlled by E‐Prime 2. In all tasks, each trial began with a fixation (a red cross lasting 1 s), followed by the presentation of the search array; participants had to scan the visual field, looking for visual targets presented among distractors of the same size. Participants were instructed to respond as accurately and as quickly as possible. After a response, a black screen was presented for 1 s before the start of the next trial. The examiner visually checked that the patients' eyes were directed to the fixation before presenting a new trial.

In the EF test, participants had to locate the target (the letter "F") among the distractors (letters "E") as quickly as possible, signaling the presence or absence of the target by pressing two keys of a computer keyboard. Twenty trials were presented, 16 with the target and four without. In the Triangles test, the participant had to scan the visual array and verbally report the number of targets (yellow triangles) among distractors (yellow squares), pressing the space bar to indicate the end of the scan. Twenty trials were administered.

#### Vision‐related ADLs

A 10‐item questionnaire [23, 35], assessing the most frequent visual difficulties experienced by patients with HVFDs, was administered. Participants rated on a 5‐point scale (from 0 = no problem to 4 = very frequent and relevant problem) to what extent they experienced difficulties in 10 ADLs. The total score range was 0–40.

### Analyses

All analyses were carried out with jamovi 2.5. Alpha was always set at 0.05.

By means of Kruskal–Wallis tests, we first compared tDCS groups in terms of clinico‐demographic features (i.e., age, disease duration, visual field size), baseline performance in each test, as well as the extension of the lesion of brain areas and white matter tracts.

To assess tDCS effects during the AVT, the percentage of accuracy (ACC) in each daily session was analyzed via a repeated measures analysis of variance (rmANOVA) with the between‐subject factor tDCS Group (occipital, parietal, and sham tDCS) and the within‐subject factors Hemifield (blind, sighted) and Day of Training (days 1–10).

To assess tDCS effects, patients' performance (ACC and reaction/response times [RTs]) on each test was analyzed by means of rmANOVAs, with tDCS Group as between‐subject factor and Timepoint (Pre, Post, FU1, FU4) as within‐subject factor.

Bonferroni‐corrected post hoc tests were run in the case of significant interactions. Moreover, for each timepoint, we computed post‐treatment changes [(Post − Pre)/Pre x 100] and looked for their association with baseline performance on each test, clinico‐demographic variables, and visual field size, as well as lesion extension of cerebral lobes and white‐matter tracts by means of Spearman correlations. Significant predictors were used in separate general linear models (GLMs) with tDCS Group as factor.

## RESULTS

The comparisons of tDCS groups at baseline, before the treatment, did not show between‐group differences with respect to age, disease duration, visual field size (all *p* > 0.45), performance on every tests (all *p* > 0.28), lesion volume and its extension at the level of lobes, areas, and tracts (all *p* > 0.16; see Table 2).

**TABLE 2:** Between‐group comparisons of clinical data at baseline, before the treatment.

Baseline (pre‐treatment) data	Occipital tDCS	Parietal tDCS	Sham tDCS	*p* ^a^
Age, years	51.7 ± 14.2	54 ± 15.3	56.2 ± 11.7	0.85
Disease duration, days	225 ± 84.2	604 ± 823	273 ± 102	0.74
Visual field size^b^	−11.7 ± 6.94	−10.5 ± 2.44	−11.7 ± 3.53	0.87
Lesion volume, cm^3^	39.9 ± 21.4	32.8 ± 19.2	36.7 ± 51.2	0.45
Visual detection with eye movements, ACC, %				
Blind hemifield	41.4 ± 15.5	48.4 ± 16.6	54 ± 12.1	0.28
Sighted hemifield	96.8 ± 3.8	91.7 ± 6.2	87.2 ± 7.6	0.06
EF test				
RTs, ms	5106 ± 2168	4827 ± 2057	4244 ± 1523	0.78
ACC, %	86 ± 7.6	80.3 ± 9.9	82.6 ± 17.7	0.56
Triangles test				
RTs, ms	7713 ± 2188	8427 ± 2549	7169 ± 2059	0.68
ACC, %	69.4 ± 17.1	66.9 ± 12.6	68.2 ± 11.2	0.99
ADLs	13.2 ± 6.71	10.7 ± 6.12	11.2 ± 8.66	0.80

*Note*: Mean values ± SD are reported.

Abbreviations: ACC, accuracy; ADLs, vision‐related activities of daily living questionnaire; RT, reaction/response time; tDCS, transcranial direct current stimulation.

^a^ Kruskal–Wallis tests.

^b^ Average mean deviation (in decibel, db) of Humphrey's monocular visual field analysis of the left and the right eye, 30‐2, Swedish Interactive Thresholding Algorithm standard.

**FIGURE 3:**
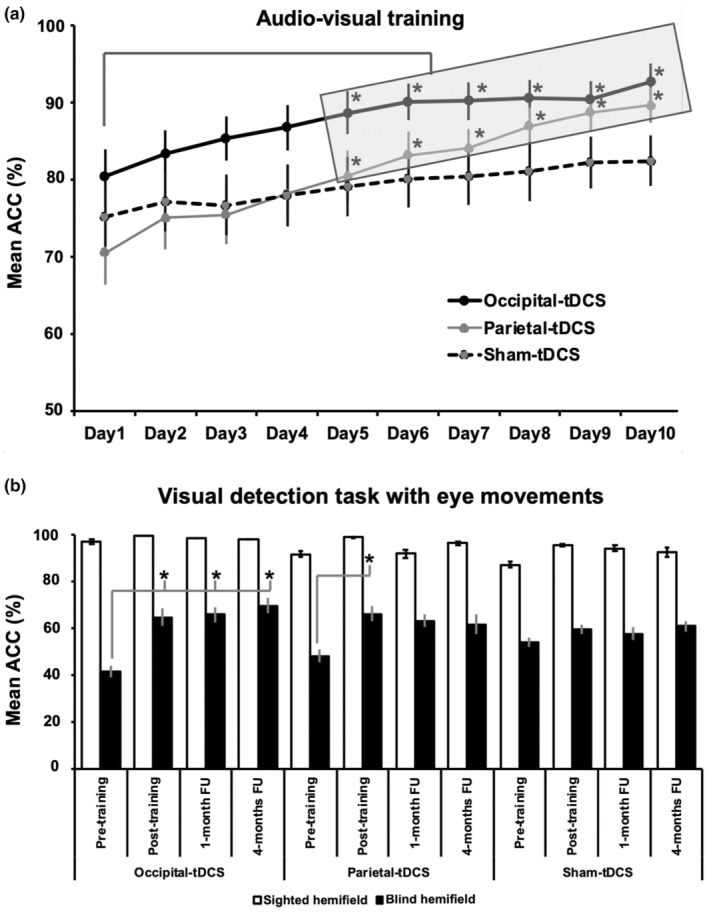
Improvements during the audiovisual training (AVT) and in the visual detection task with eye movements. (a) Performance during the AVT, showing mean percentage of audiovisual detection accuracy (ACC) in the whole visual field on each day of the 2‐week AVT (1st week = day 1–5, 2nd week = day 6–10), representing the significant 'Day × tDCS Group' interaction. Black lines = performance of patients who received occipital tDCS, gray lines = patients who received parietal tDCS, dotted black lines = sham tDCS. Asterisks indicate a significant improvement with respect to the 1st day of training (all *p* < 0.001). (b) Performance in the visual detection task with eye movements, showing mean percentage of visual detection AC in the sighted hemifield (white bars) and in the blind hemifield (black bars) pre‐training, post‐training, and at 1‐month and 4‐month follow‐ups (FUs) for each experimental group (occipital, parietal, and sham tDCS). Asterisks indicate a significant improvement (all *p* < 0.03) with respect to pre‐training performance. Error bars = standard error.

With respect to the daily improvements during the AVT, the rmANOVA showed significant effects of Hemifield (*F* = 65.45, *p* < 0.001), Day (*F* = 2.17, *p* < 0.007), Hemifield × Day (*F* = 18.99, *p* < 0.0001), and tDCS Group × Day (*F* = 2.17, *p* < 0.0001), whereas the factors tDCS and the interactions Hemifield × tDCS Group and Hemifield × tDCS Group × Day did not reach the significance level (all *F* < 1.42, all *p* > 0.13). The tDCS Group × Day interaction showed that real tDCS over the occipital or parietal cortex increased visual detections in both hemifields starting from the 5th day of AVT, as compared to the 1st day (Figure 3a; occipital tDCS: day 1 = 80% vs. day 5 = 89%, day 6 = 90%, day 7 = 90%, day 8 = 91%, day 9 = 91%, day 10 = 93%, all *p* < 0.03; parietal tDCS: day 1 = 71% vs. day 5 = 80%, day 6 = 83%, day 7 = 84%, day 8 = 87%, day 9 = 89%, day 10 = 90%, all *p* < 0.001). With sham tDCS, no significant changes occurred across days of training (all *p* > 0.2). The Hemifield × Day interaction showed a gradual increase in visual detections in every group only in the affected hemifield (from day 1 = 56% to day 10 = 80%, all *p* < 0.01), but not in the intact hemifield (from day 1 = 95% to day 10 = 97%, *p* = 0.9), regardless of the tDCS group, hence showing that, at least during the AVT, audiovisual detection accuracy in the blind and intact hemifield was similar in the three tDCS groups. Other comparisons did not reach the significance level (all *F* < 1.4, *p* > 0.1).

With respect to the visual detection task with eye movements, significant effects were found for Hemifield (*F* = 103.8, *p* < 0.00001), Timepoint (*F* = 13.24, *p* < 0.00001), Hemifield × Timepoint (*F* = 5.72, *p* = 0.002), and tDCS Group × Hemifield × Timepoint (*F* = 2.68, *p* = 0.03); no significant effects were found for the factor tDCS Group and the interactions Hemifield × tDCS and Timepoint × tDCS Group (all *F* < 0.89, *p* > 0.5). The significant tDCS Group × Hemifield x Timepoint interaction showed a significant improvement in visual detections in the contralesional hemifield immediately after the training, stable at the follow‐ups, in patients who received the real occipital tDCS (baseline = 41% vs. post‐training = 65%, FU1 = 66%, FU4 = 70%, all *p* < 0.0003). With parietal tDCS, visual detections increased at the end of treatment (baseline = 48% vs. post‐training = 66%, *p* < 0.03), but patients' performance did not differ from baseline at the follow‐ups (FU1 = 63%, FU4 = 61%, all *p* > 0.1). With sham tDCS, no significant changes emerged (baseline = 54% vs. post‐training = 60%, FU1 = 58%, FU4 = 61%, all *p* > 0.9). Before the AVT, visual detection accuracy with eye movements in the affected hemifield did not differ across groups (occipital tDCS, pre‐training = 41% vs. parietal tDCS = 48% vs. sham tDCS = 54%, all *p* > 0.5), and the post‐training improvements were not different in the three groups (all *p* > 0.8).

In all groups, no post‐training improvements were found in the ipsilesional hemifield (all *p* > 0.4; see Figure 3b).

**FIGURE 4:**
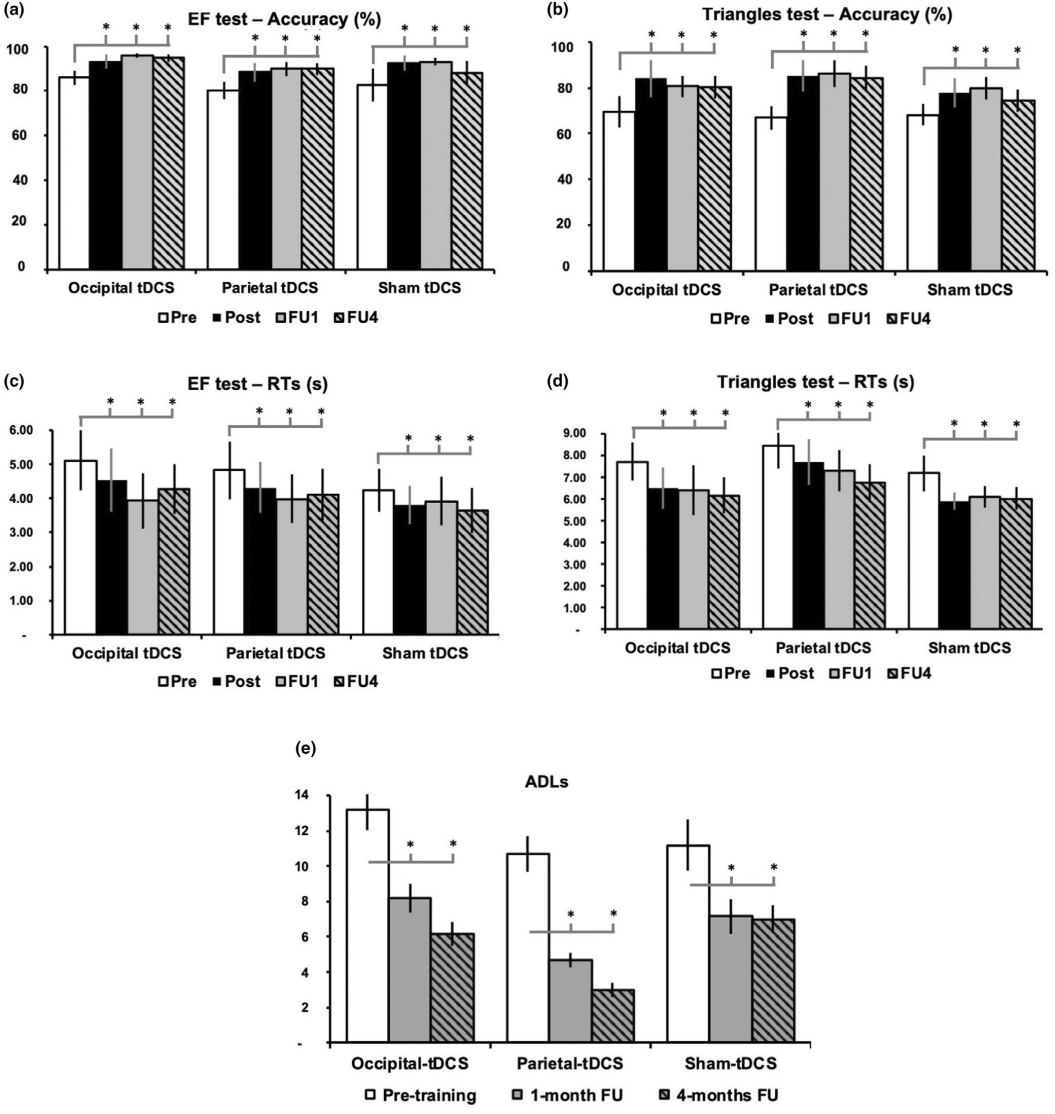
Visual search tests and questionnaire about vision‐related activities of daily living (ADLs; e). (a–d) Mean accuracy as a percentage (a, b) and response times (RTs; c, d) for the EF and the Triangles tests. White bars = pre‐training, black bars = post‐training, gray bars = follow‐up at 1 month (FU1), striped black/gray bars = follow‐up at 4 months (FU4). Error bars = standard error. Asterisks indicate a significant improvement with respect to pre‐training. tDCS, transcranial direct current stimulation.

With respect to the EF and Triangles tests, the rmANOVAs on accuracy showed the effect of Timepoint (all *F* > 10.12, all *p* < 0.001), showing post‐training improvements still observable after 4 months (all *p* < 0.02; see Figure 4a,b). Likewise, for RTs (Figure 4c,d), we found a significant effect of Timepoint (all *F* > 11.21, all *p* < 0.001); patients were faster after the AVT, with benefits persisting up to FU4 (all *p* < 0.03). No significant effects of tDCS Group (all *F* < 0.75, all *p* > 0.5) or tDCS Group × Timepoint interactions (all *F* > 1.32, all *p* > 0.3) were observed.

Finally, the analysis of ADLs questionnaire (see Figure 4e) revealed only a main effect of Timepoint (*F* = 22.96, *p* < 0.0001), but not of tDCS Group (*F* = 0.63, *p* = 0.5) and tDCS Group × Timepoint (*F* = 0.63, *p* = 0.6); functional improvements emerged at the follow‐ups in all groups (Pre = 11.7 vs. FU1 = 6.7 and FU4 = 5.4, all *p* < 0.0001).

Correlation analyses showed that the baseline performance in the visual detection task with eye movements was associated with post‐training improvements at all timepoints (all *r* < −0.58, all *p* < 0.014); the treatment‐induced improvement was greater with poorer performance at baseline. These effects, still observed in GLMs at FU1 (*β* = −0.46, *p* = 0.03) and FU4 (*β* = −0.47, *p* = 0.04), were independent from the application of real or sham tDCS at all timepoints considered (*F* > 2.79, *p* < 0.1). A similar effect of the baseline performance was found for the accuracy improvement in the EF test immediately after the treatment (*β* = −0.79, *p* < 0.001) and at FU1 (*β* = −0.58, *p* < 0.001), where an effect of lesion volume was also observed (*β* = 0.44, *p* = 0.005), suggesting that participants with bigger lesion volumes improved the most. More specifically, the involvement of the lesioned parietal cortex predicted EF accuracy improvements at FU1 (*β* = 0.73, *p* = 0.02) and FU4 (*β* = 0.77, *p* = 0.02). Across models, no differences between tDCS groups were detected (all *F* < 3.6, all *p* > 0.07). The baseline performance also predicted accuracy improvements on the Triangles test at FU1 (*β* = −0.60, *p* = 0.009) and FU4 (*β* = −0.53, *p* = 0.02). These improvements were also associated with more extended damage to the inferior fronto‐occipital fasciculus (FU1; *β* = 0.78, *p* = 0.012) and the optic radiations (FU4; *β* = 0.661, *p* = 0.03). No effects of tDCS Group were observed (all *F* < 2.15, all *p* > 0.15). Age, disease duration, and visual field size, as measured by computerized perimetry, were not associated with post‐treatment improvements across all the analyses (all *p* > 0.18).

## DISCUSSION

The main findings of this proof‐of‐principle study are: (i) faster improvements during the AVT when anodal tDCS is applied to ipsilesional occipital and parietal areas during training; (ii) post‐training improvement of visual detections with eye movements in the blind hemifield when tDCS is applied, with long‐lasting effects when targeting the occipital cortex and short‐lasting effects when targeting the parietal cortex; (iii) no boosting effects of tDCS on AVT‐induced improvements in visual search and ADLs.

The first important result is the daily improvement during AVT, where patients showed enhanced progress with the adjuvant effect of occipital and parietal tDCS compared to AVT combined with sham tDCS. This accelerated improvement, evident from the end of the first week of training, highlights the potential for shortening rehabilitation times and increasing therapeutic adherence, given that visual field rehabilitation is typically long and intensive. The 'whole‐field' improvements in visual detections with eye movements align with previous findings showing that HVFDs impair scanning and visual exploration (e.g., elevated search times, numerous and long fixations), affecting both the blind and intact hemifield [6, 36, 37].

The power of tDCS to modulate synaptic plasticity likely explains these positive effects on visual learning and recovery as observed in both animal and human studies [13]. The neuromodulatory nature and the low spatial resolution of tDCS make it suitable for its use during compensatory visual scanning therapy, modulating activation and connectivity beyond the stimulated site, that is, interacting with the ongoing brain activity (i.e., state‐dependency [38] of the networks activated by AVT). Stimulating heteromodal parietal areas may facilitate audiovisual interactions [39, 40, 41] and improve the deployment of cross‐modal spatial attention and orienting [33], while occipital stimulation likely enhances perceptual processing of visual signals from the affected hemifield (e.g., Sabel et al. [13], Antal et al. [42, 43]), leading to improved oculomotor responses.

Interestingly, at the end of treatment, anodal occipital tDCS combined with AVT significantly improved visual detections with eye movements in the blind hemifield, with these effects persisting for at least 4 months, as compared to AVT with sham tDCS. Parietal tDCS also improved visual detections but only in the short term, suggesting an attention‐mediated mechanism following the neuromodulation of the posterior parietal areas rather than a perceptual enhancement. The long‐term improvements with occipital tDCS may reflect increased activity in ipsilesional visual areas, functionally similar to the perceptual improvements obtained with restorative trainings combined with occipital tDCS [18, 19, 20]. Hence, we propose that excitatory occipital neuromodulation may reactivate perilesional areas, enhancing visual input from the blind hemifield and facilitating eye movements toward contralesional visual events when eye movement are allowed [18, 19, 20]. In other words, the stable improvement of visual detection in the blind hemifield induced by the occipital tDCS would reflect perilesional plasticity and greater sensitivity of the ipsilesional visual areas, whereas the short‐lived facilitation of visual detection by the parietal tDCS is more likely mediated by an enhanced ability to deploy visuospatial attention toward stimuli in the blind hemifield.

Another possible mechanism that could explain the tDCS effects both during the training and at its end is the strengthening of multisensory circuits involved in blindsight (proposed as main mechanisms activated by AVT [26, 44, 45, 46, 47]), which could support saccadic movements toward the blind field and improve visual awareness. This aligns with the work by Matteo and colleagues [19], who combined anodal tDCS of ipsilesional parieto‐occipital areas with a blindsight training in two patients with HVFDs, obtaining an increase in the detection of visual stimuli presented in the periphery of the blind hemifield (without eye movements to the targets, at variance with our task) along with a reduction of vision‐related disability in ADLs.

In addition to the benefits of combined neuromodulation and AVT, our study reaffirms the efficacy of this training in improving visual scanning and reducing disability in ADLs, with long‐term benefits persisting for at least 4 months [25]. The benefits in ADLs likely result from a progressive consolidation of oculomotor strategies, leading to faster responses to stimuli from the blind side and more effective exploration of the whole visual scene. Although some studies showed lasting benefits from a single training course (up to 5 years post‐training [11]), repeated training may further enhance long‐term retention of these effects, potentially facilitated through, for example, home‐based telerehabilitation [20]. Our findings are consistent with previous literature supporting the effectiveness of compensatory multisensory treatments even for chronic acquired HVFDs throughout the lifespan [22, 23, 25, 48]. Finally, AVT may be particularly beneficial for individuals with severe visuospatial impairments and extensive parieto‐occipital damage, who are less likely to develop compensatory strategies independently [6, 49].

Although promising, our results refer to a proof‐of‐principle study in a small, quite heterogenous, sample of patients, in which baseline clinical variability is evident; this constitutes the main limitation of this study, highlighting the need of further randomized controlled trials with larger samples and by adopting a within‐subjects design, also in light of the role of pre‐treatment visual impairment in the success of treatment, as suggested by our correlation analyses. Future studies should also investigate the potential restorative effects of occipital tDCS combined with AVT, exploring whether this approach can lead to visual field enlargement by reactivation of perilesional areas [13]. To this aim, a computerized visual field perimetry should be repeated after the training. Future studies are also needed to clarify the mechanisms facilitated by the neuromodulation of parietal and occipital areas during the AVT and the possible role of blindsight.

In conclusion, our proof‐of‐principle study demonstrates, for the first time, that the compensatory AVT for HVFDs benefits from concomitant neuromodulation of the occipital and parietal areas. Occipital tDCS, in particular, can induce longer lasting effects on visual processing in the blind hemifield, even in the chronic stage of illness. These results encourage further investigations to optimize compensatory visual rehabilitation using neuromodulatory techniques, and to identify responders and nonresponders to the treatment. Above all, our results point to new opportunities for recovery in patients with HVFDs, offering avenues to improve daily functioning and quality of life.

## FUNDING INFORMATION

This work was funded by Italian Ministry of Health ‐ Ricerca Corrente.

## CONFLICT OF INTEREST STATEMENT

The authors declare no conflict of interest.

## Supporting information


Appendix S1.

## Data Availability

The datasets analyzed in this study can be found on Zenodo at https://doi.org/10.5281/zenodo.11619409. This set of raw data is accessible only by request, because it includes sensitive information. Please address your request to the corresponding authors.
