# Supplementary material for: Enhancing multisensory rehabilitation of visual field defects with transcranial direct current stimulation: A randomized clinical trial
Source: Eur J Neurol. 2024 Nov 28;32(1):e16559. doi: 10.1111/ene.16559 (PMC11625917; doi:10.1111/ene.16559)
Supplement: Supplementary file 1 — Appendix S1. [file ENE-32-e16559-s001.docx]

Supplemental Material

**Sample size estimation by means of power analysis**

The sample size was calculated with G*Power (3.1.9.6; Heinrich-Heine-Universität Düsseldorf, Germany) for a mixed-ANOVA considering: 1-beta=.08, alpha=.05, a medium effect size (f=0.25), 4 measurements (i.e., 4 timepoints), 3 groups (i.e., occipital tDCS, parietal tDCS, sham tDCS), a strong correlation between repeated measures of 0.75, and sphericity correction=1. The analysis indicated a minimum sample size of 18.

**Lesion Mapping Procedure and results**

Brain lesions were first delineated from MRI scans in MRIcroGL^1^. Brain scans and lesion maps were normalized (function “MR segment-normalize”) onto an age-appropriate template by means of the Clinical Toolbox^2^ for Statistical Parametric Mapping (SPM12 ^3^) in MATLAB 2019b (The MathWorks Inc., 2019).

The mean lesion volume was 36.5 cm^3^ ± 31.5 (range = 3.95-127 cm^3^). According to the Automated Anatomical Labelling atlas (AAL ^4^), the most affected areas, irrespective of the lesion side, were: the calcarine sulcus (N=15), the lingual gyrus (N=15), the superior (N=12), the middle (N=12), and the inferior (N=11) occipital lobes, as well as the cuneus (N=9), and the fusiform gyrus (N=12).

Moreover, we calculated the overall lesion extension (i.e., number of voxels) of the occipital, temporal, and parietal lobes by summing the lesion extension of single areas ^5^. The occipital lobe included the calcarine sulcus, the cuneus, the lingual gyrus, the superior, middle, and inferior occipital gyrus; the temporal lobe included the inferior, middle, and superior gyri, as well as the fusiform area; for the parietal lobe, we included the post-central gyrus, the inferior and superior parietal lobules, the supramarginal gyrus, the angular gyrus, and the precunues. Lastly, we calculated a temporo-parietal lesion burden, as the sum of lesion extension of parietal and temporal areas, excluding the more ventral fusiform gyrus.

Following a second co-registration on an MNI-152 template (slices thickness = 1 mm), the lesion maps were processed with Tractotron in Brain Connectivity Behaviour Toolkit (<http://toolkit.bcblab.com/> ^6^) to obtain a probabilistic damage quantification of major white matter fibers. Lesion extension (i.e., the proportion of tract affected by the lesion) were considered of those tracts with a lesion probability > 50 %. A number of intra- and inter-hemispheric white matter bundles were affected: corpus callosum (N=15), inferior fronto occipital fasciculus (IFOF; N=15), inferior longitudinal fasciculus (N=15), optic radiation (N=14), posterior cingulum bundle (N=12), the 1^st^ and 2^nd^ branches of the superior longitudinal fasciculus (SLF; N=9).

**References**

1. Rorden C, Brett M. Stereotaxic display of brain lesions. *Behav Neurol*. 2000;12(4):191-200. doi: 10.1155/2000/421719.

2. Rorden C, Bonilha L, Fridriksson J, Bender B, Karnath H. Age-specific CT and MRI templates for spatial normalization. *Neuroimage*. 2012;61(4):957-965. doi: 10.1016/j.neuroimage.2012.03.020.

3. Penny WD, Friston KJ, Ashburner JT, Kiebel SJ, Nichols TE. *Statistical parametric mapping: The analysis of functional brain images.* Elsevier; 2011.

4. Tzourio-Mazoyer N, Landeau B, Papathanassiou D, et al. Automated anatomical labeling of activations in SPM using a macroscopic anatomical parcellation of the MNI MRI single-subject brain. *Neuroimage*. 2002;15(1):273-289. doi: 10.1006/nimg.2001.0978.

5. Diana L, Casati C, Melzi L, Bianchi Marzoli S, Bolognini N. The effects of occipital and parietal tDCS on chronic visual field defects after brain injury. *Front Neurol*. 2024;15:1340365. doi: 10.3389/fneur.2024.1340365.

6. Foulon C, Cerliani L, Kinkingnehun S, et al. Advanced lesion symptom mapping analyses and implementation as BCBtoolkit. *Gigascience*. 2018;7(3):1-17. doi: 10.1093/gigascience/giy004.
